# Diphtheria

**Published:** 1911-03

**Authors:** William F. Waugh

**Affiliations:** Head of the Department of Therapeutics, Loyola University (Bennett Medicine College), Chicago


For Texas Medical Journal.

### Diphtheria.

BY WILLIAM F. WAUGH, M. D., A. M., HEAD OF THE DEPARTMENT OF THERAPEUTICS, LOYOLA UNIVERSITY (BENNETT MEDICINE COLLEGE), CHICAGO.

If there is a name in the full category of human plagues that blanches the mother's cheek and sends a thrill of fear through the heart of even the grilled old doctor, it is diphtheria. Many be the conflicts we have had with this terrible killer, battles royal every one, and, while the victory has perched on our banner many a time, there have been other times when we have been humbled to the dust by the doughty bacillus whose identity Klebs and Loeffler revealed. Yes, truly; despite our utmost efforts we have lost out, and every particle of conceit has long since disappeared before this malady.

I trust no sore throat. Despite the most perfect evidence that our case is not true diphtheria today, we dare not assert that it will not be diphtheria tomorrow. But this does not excuse one for calling everything tonsilar diphtheria, or subjecting all to the now classic treament by antitoxin. This wonderful remedy has its place, but this is strictly in dealing with true diphtheria.

The diagnosis must usually be clinical, as bacteriology is too slow to be aught but confirmatory. A red throat is rarely diphtheritic; a primary membrane is always diphtheria. The membrane appears on the tonsils, soft palate, faucial pillars, uvula or pharynx, is white or gray, tough and adherent.

A pultaceous exudate resembles curdled milk, neither coheres nor adheres, and breaks down in water. It is not diphtheria. Necrosis attends Vincent's angina; the deposit is greenish-gray and thready and rests on an ulcer, generally tonsilar and unilateral. It is not diphtheria.

Follicular tonsilitis is ushered in with high fever, bone-ache and decided general symptoms, with white spots, five to ten, on each side, remaining unaltered. If diphtheritic, they spread and thicken. They are not membranous.

Herpetic angina is a misnomer. The spots are very small, round grayish-white, with a red areola. Vesicles may appear.

In follicular cases there is glandular hypertrophy with granulations on the posterior wall of the pharynx, etc., forming little abscesses. The application of chemical cauterants may give these the appearance of true diphtheria. But if the exposure to diphtheria has been direct, it is safe to so diagnose these. Laryngeal implication speaks for the more serious malady. Coryza is ominous, especially if unilateral and bloody, or followed by erosion of the nares. Cervical adenitis also points to diphtheria, if marked.

The practice of pronouncing all these cases diphtheria subjects the parents to needless terrors; the indiscriminate application of antitoxin supplies a peril of its own. There is no objection to the use of local germicides, which cure all these pseudo-diphtherias, and prevent the development of the real disease. Loeffler's solution is good; strong salt water is by no means valueless; saturated solution of salicylic acid exceedingly effective, especially against scarlatinal angina; but best of all is the solution of nascent chlorine, made by mixing a dram each of potassium chlorate and strong hydrochloric acid, and adding four ounces of water.

Meanwhile, do not forget to empty and disinfect the bowels, to saturate the patient's system with calcium sulphide, to clean up the house and neighborhood, and to watch unremittingly for the first definite sign of real diphtheria, when antitoxin should be administered.

